# Prevalence, clinical presentation, management of pituitary tumour and its complications among elderly population in Mumbai, India

**DOI:** 10.6026/973206300191139

**Published:** 2023-12-31

**Authors:** Kumar Abhinav, Uday B Andar

**Affiliations:** 1 Department of Neurosurgery, Lilavati Hospital and Research Centre, Mumbai, India; 2 Department of Neurosurgery, BaiJerbai Wadia Hospital for Children, Mumbai, India

**Keywords:** Pituitary tumours, clinical presentation, management, complications, elderly

## Abstract

Pituitary tumour is not typically thought of as an elderly patient's condition. Hence, we examined all cases of confirmed or suspected pituitary tumour diagnosed in a tertiary hospitals at Mumbai, India during May 2015 and May 2023 among patients over the age of 70 to evaluate the prevalence, clinical presentation, management, complications in elderly patients with a pituitary tumour. After the age of 70 years, 16% people having pituitary tumour were observed. The volume of fossa was statistically greater in elderly patients. The duration of follow up was statistically longer in younger controls. The visual defects observed in elderly group were greater than young patients. Pituitary adenomas in old patients can be treated with trans-sphenoidal-adenomectomy. However, the proportion is lower than younger controls. Data shows that post-operative radiotherapy was more commonly observed in old patients with pituitary adenoma than younger controls.

## Background:

The clinical characteristics of pituitary tumour are complicated and varied, and they exhibit a wide spectrum of proliferating and invasive behaviours [1-2]. While some aggressive pituitary tumours (PAs) grow quickly and are resistant to standard therapies, other aggressive PAs are asymptomatic and maintain their size for a long time [3-4]. While some pituitary tumours are huge, invasive adenomas that do not exhibit excessive hormone release, others are small pituitary tumours that exhibit significant systemic metabolic problems brought on by excessive pituitary production of hormones [5, 6]. Adenomas, or pituitary tumours, are frequent neoplasms that affect more than seventeen percent of Americans [7-8]. Physical and metabolic alterations are caused by pituitary adenomas. The symptoms that are felt can vary depending on the adenoma's size. Neonasal discharge, vomiting, vertigo, migraines, vision problems, a running nose (CSF leakage), disorientation, and convulsions are some of the general as well as physical signs and symptoms [9-10]. Certain forms of pituitary tumours are accompanied by particular indications and symptoms. The pituitary gland and various parts of the brain are close together, hence adenoma-associated morbidities such stroke, loss of vision, meningitis and CSF leakage, and cerebral palsy are common [10-11]. The outcome for a pituitary adenoma with an early diagnosis can be favorable, with a probability of less than one percent death rate following total tumour removal operation [12-13].

There are two competing hypotheses for the cause of pituitary tumours. Pituitary adenomas were thought to be inherent irregularities in the gland alone in one idea, and constantly triggered by a hormone or other factor from the hypothalamus in another theory [14-15]. But modern genetic technology has demonstrated that pituitary tumours develop from a single cell alteration accompanied by clonal proliferation. Pituitary neoplasm is a multi-step process that involves abnormal hormone synthesis, differentiation of cells, and growth [16- 17]. Many pituitary tumours are genetically idiopathic, but others are connected to multiple endocrine neoplasia form 1 (MEN1). MEN1 can lead to the pituitary gland, among other endocrine glands, becoming overactive or enlarging [18-19]. Carney complex, another hereditary factor causing pituitary adenomas, is characterised by tumours of connective tissue as well as endocrine gland tumours as well as patchy skin colouring [20-21]. Each of the disorders mentioned above has the potential to result in pituitary tumours, but other elements must also be taken into account, such as advancing age and specific hereditary conditions, which may put individuals at a greater danger of getting converted into pituitary tumours [21- 22]. The second most frequent primary brain tumour in humans, pituitary tumours make for to ten to fifteen percent of all intracranial tumours. The projected incidence of PAs was determined to be 16.7% overall (14.4% in autopsy analyses and 22.5 percent in radiologic investigations) in a meta-analysis of radiological assessment and autopsy research [23- 24]. In the past ten years, there has been a noticeable rise in the frequency of PAs due to the widespread use of magnetic resonance imaging (MRI). Pituitary tumour is rarely included in geriatric medicine textbooks, and they are not typically thought of as an elderly patient's condition [1-4]. The widely recognized endocrine disorders brought on by the functional pituitary adenomas' related hormone excessive production typically appear earlier in life. The common physical and hormonal alterations of ageing, as well as the prevalence of various illnesses, may make it more difficult to diagnose pituitary dysfunction in the elderly [5,8]. The literature has paid little consideration to the presentation and treatment of pituitary tumour in the elderly. Previous research either involved post-mortem cases or only a restricted number of older people [11, 14]. Therefore, it is of interest to analyze all of these cases that presented to a Neurosurgical Centre during an eight-year period in order to evaluate the prevalence, clinical presentation, management, complications in elderly patients with a pituitary tumour.

## Materials and Methods:

We examined all cases of confirmed or suspected pituitary tumours diagnosed in tertiary hospitals during May 2015 and May 2023 in patients over the age of 70. Our goals were to assess the safety and effectiveness of trans-sphenoidal surgery for older patients, especially those with chiasmal contraction, and to compare the different type of manifestation in the elderly versus those of a younger cohort of controls, matched for size of tumour and type of tumour.

## Patients and methods:

The endocrine case directory, hospital activity monitoring data, and the pituitary surgery record were searched to find all patients who were older than 70 at the time of their initial pituitary tumour diagnosis and who presented to or got referred to the locale-specific neurosurgical institute between May 2015 and June 2023. The first possible matching for genders, tumour dimensions, and tumour subtype was utilised to create a control cohort from a series of patients who presented during the same time period but at an age under 70. These three factors were the only ones taken into account.

The volume of pituitary fossa was determined following the Lusted and Keats methodology from plain skull radiographs for all individuals [2].To determine the size of any extra sellar tumours, these were complemented by computerized tomography utilising GE 8800 and, more currently introduced, the Siemens Somatom DR1 scanning devices. Clinical assessments of visual acuities as well as fields were made, and Goldmannpenmetry was done if vision was compromised. Dynamic assessment of adrenocortical function, serum prolactin, free or total thyroxine and gonadotrophins (including the hormone testosterone in men) were measured as part of the elderly patients' endocrine examination. The latter typically used depot synacthen or injectable glucagon, while some had carefully monitored insulin hypoglycemia tests.

The standard criteria previously published [3] were used to interpret the endocrine results. In our testing facility, the normal threshold for prolactin is < 500 mU/l for men and < 700 mU/l for women. Following surgical procedures, patients underwent further testing 4-6 weeks later and on subsequent occasions. Previous studies have described the specifics of the surgical procedure [3] and tumour profiling using immunocytochemistry along with electron microscopy [4].

## Statistical analysis:

SPSS software 2021 (IBM, USA) was used for all analyses. Median (range) was used to represent numerical variables. Two way ANOVA and Pearson coefficient analysis was carried out. A p value < 0.05 was considered statistically significant.

## Results:

## Prevalence and distribution of patients with pituitary tumors:

During the eight year retrospective evaluation records of a total of 704 patients were evaluated. After the age of 70 years, 44 (16%) people having pituitary tumours were found. Six elderly individuals having acromegaly and two patients with Cushing's syndrome were present. Among the remaining group of 36, two cases of suspected prolactinoma (serum PRL >20,000 mU/1) and two cases of surgically confirmed craniopharyngioma were observed. Finally, 32 patients had massive, nonfunctioning adenomas of the pituitary gland in which 22 patients underwent surgery. 36 younger patients with pituitary adenoma were also included in the study. Comparisons were made between the study participants having pituitary adenoma aged more than 70 years and younger patients with pituitary adenoma. The fossa volume in elderly patients was 3291 (826-7841) mm3 while it was 2981 (1225-9051) mm3 in younger patients. The supra-sellar extension was 14 (0-28) mm in elderly patients while it was 13 (0-26) mm in younger controls. Follow up in elderly patients was carried out for 22 months (2-44) in elderly patients while it was carried out for 37 (7-68) months in younger control patients. The volume of fossa was statistically greater in elderly patients. The duration of follow up was statistically longer in younger controls (Table 1).

## Clinical presentation:

The visual defects were observed in 21 patients in elderly group while visual defects were observed in 16 patients in young patients. The difference in observations was meaningful statistically (p=0.001). The neurological defects were observed in 7 patients in elderly group while it was observed in 6 patients of younger control group. The endocrine symptoms involving ACTH, TSH and gonadotrophin was observed in 2 patients of elderly patients. The endocrine symptoms were observed in 8 patients in younger controls. The difference in observations was meaningful statistically (p=0.001). The incidental finding was observed in five patients in elderly group while it was four in younger controls. Pituitary apoplexy was observed in one elderly patient while it was observed in two younger controls (Table 2).

## Management and complications:

Trans-sphenoidal-adenomectomy was carried out in 24 patients among elderly patients while 30 patients in younger controls underwent this treatment procedure. The difference in findings was meaningful statistically. Post-operative radiotherapy was carried out in 2 elderly patients while no younger control underwent this treatment procedure. Shunt for hydrocephalus and endocrine replacement was carried out in 2 elderly patients while it was carried out in 6 younger controls. Tumour biopsy and radical radiotherapy was carried in 2 younger controls while no such treatment was carried out in elderly patients. Endocrine replacement only was carried out in 06 elderly patients while no procedure was carried out in younger control. No specific treatment was carried out in four elderly patients (Table 3).

## Discussion:

Pituitary tumours have complex and diverse clinical traits and display a wide range of proliferative and invasive tendencies. Other aggressive pituitary tumours are asymptomatic and maintain their size for a long time, whilst some aggressive PAs grow quickly and are resistant to conventional therapy [12-13]. Some pituitary tumours are large, invasive adenomas that have localized mass effects but do not release excessive amounts of hormones, whereas others are small pituitary tumours that cause serious systemic metabolic issues as a result of excessive hormone production in the pituitary [14-15].

Pituitary tumours are seldom discussed in geriatric medicine texts and aren't frequently associated with senior patients [16]. The well-known endocrine conditions caused by functional pituitary adenomas' excessive hormone production often manifest earlier in life. It may be more challenging to diagnose pituitary dysfunction in the elderly due to the usual physical and hormonal changes of ageing, as well as the frequency of several diseases [17- 18]. The presentation and treatment of pituitary tumours in the elderly have received less attention in the literature [19-20]. In order to assess the way of presentation, endocrine disruption, medication, and outcomes for older individuals with a pituitary tumour, we reviewed all of these cases that presented to a Neurosurgical Centre over an eight-year period.

In this study during the eight year retrospective evaluation records of a total of 704 patients were evaluated. After the age of 70 years, 44 (16%) people who presented with pituitary tumours were found. Out of 44, six elderly individuals having acromegaly and two patients with Cushing's syndrome were present. Among the remaining group of 36, two cases of suspected prolactinoma (serum PRL <20,000 mU/1) and two cases of surgically confirmed cranio-pharyngioma were observed. Finally, 32 patients had massive, nonfunctioning adenomas of the pituitary gland in which 22 patients had surgery after being diagnosed with immunologically. The fossa volume in elderly patients was 3291 (826-7841) mm3 while it was 2981 (1225-9051) mm3 in younger patients. The supra-sellar extension was 14 (0-28) mm in elderly patients while it was 13 (0-26) mm in younger controls. Follow up in elderly patients was carried out for 22 months (2-44) in elderly patients while it was carried out for 37 (7-68) months in younger control patients. The volume of fossa was statistically greater in elderly patients. The duration of follow up was statistically longer in younger controls.

The clinical characteristics of these malignancies in the elderly have received little attention, and individuals over 70 are the exception rather than the rule in most series. One of 47 individuals having pituitary tumours and hyper-prolactinemia, for instance, was only over 70 years old when they were diagnosed, according to Schlechte et al. [6]. In a retrospective review of 111 intracranial neoplasms in individuals over 65 who were examined at a regional clinic over a period of ten years, Godfrey and Caird [7] identified just six pituitary tumours-all detected accidentally. In the American national survey of brain tumours, a greater percentage was discovered; in a period of two years where 270 of 1970 adenomas (14%) happened in patients over the age of 65 [8]. In contrast, post-mortem examinations frequently reveal pituitary adenomas.

According to Kovacs *et al.* [9], an aggregate of 22 adenomas were detected in the pituitaries taken from 1 52 men and women who were over 80 years old during unselected necropsies. Nineteen of the aforementioned were microadenomas, but two more took up the majority of the anterior part of the lobe but were never detected while the patient was alive. However, there was no proof that hyper prolactinaemia had existed throughout life in any of the nine out of the seventeen adenomas that immuno-stained for prolactin. It's interesting to note that as people age, certain strains of rats, in particular, typically acquires prolactin-secreting adenomas [10]. However, young people's necropsy studies also show a high prevalence of pituitary microadenomas, which most frequently immuno-stain for prolactin [11]. Twelve of the 24 patients with craniopharyngiomas who were older than 40 years old who were described by Russell and Pennybacker [15] had memory impairment, and eight had dementia. These symptoms, which are typical of the aged population, were attributed to hippocampal impairment or hydrocephalus with obstruction.

Adenomas, also known as pituitary tumours, are a common neoplasm that affects more than 17% of Americans. Pituitary adenomas result in physical and metabolic changes. Depending on the size of the adenoma, different symptoms could be experienced [21, 22]. Some of the general as well as physical indications and symptoms include nasal discharge, vomiting, vertigo, migraines, vision issues, a runny nose (CSF leakage), disorientation, and convulsions. Specific signs and symptoms are associated with specific types of pituitary tumours [17, 19]. Because of the proximity of the pituitary gland to various areas of the brain, adenoma-related morbidities such stroke, visual loss, meningitis and CSF leaking, and cerebral palsy are frequent [14-17].

A pituitary adenoma with an early diagnosis may have a fair prognosis, with a likelihood of less than 1% following total tumour removal.For the origin of pituitary tumours, there are two opposing theories [18-20]. In one view, pituitary adenomas were believed to be innate anomalies underlying the gland alone, while in another, they were believed to be regularly triggered by a hormone or other substance from the hypothalamus [21- 22.^23^]. But contemporary genetic technology has shown that clonal growth and a single cell change lead to the development of pituitary tumours. Pituitary neoplasm development comprises several stages, including aberrant hormone synthesis, cell differentiation, and proliferation [15-17]. Pituitary tumours can cause other endocrine glands, including the pituitary gland, to become overactive or enlarge. Many pituitary tumours are genetically idiopathic, but some are linked to multiple endocrine neoplasia [18- 19].

The visual defects were observed in 21 patients in elderly group while visual defects were observed in 16 patients in young patients. The difference in observations was meaningful statistically (p=0.001). The neurological defects were observed in 7 patients in elderly group while it was observed in 6 patients of younger control group. The endocrine symptoms involving ACTH, TSH and gonadotrophin was observed in 2 patients of elderly patients. The endocrine symptoms were observed in 8 patients in younger controls. The difference in observations was meaning statistically significant (p=0.001). The incidental finding was observed in five patients in elderly group while it was four in younger controls. Pituitary apoplexy was observed in one elderly patient while it was observed in two younger controls. Due to increasing use of magnetic resonance imaging (MRI), there has been a substantial increase in the frequency of PAs over the past ten years. Pituitary adenomas caused by the Carney complex, another genetic component, are marked by connective tissue tumours, endocrine gland tumours, and patchy skin coloration [20-24, 25]. Each of the aforementioned illnesses has the potential to develop into a pituitary tumour, but other factors must also be considered, such as ageing and specific hereditary conditions, which may increase a person's risk of developing a pituitary tumour [21-24]. Pituitary tumours account for ten to fifteen percent of all intracranial neoplasms and are the second most common primary brain tumour in adults. In a meta-analysis of radiographic evaluation and autopsy research, the estimated incidence of PAs was found to be 16.7% overall (14.4% in postmortem studies and 22.5 percent in radiologic investigations) [24-26].

Trans-sphenoidal-adenomectomy was carried out in 24 patients among elderly patients while 36 patients in younger controls underwent this treatment procedure. The difference in findings was meaningful statistically. Post-operative radiotherapy was carried out in 2 elderly patients while no younger control underwent this treatment procedure. Shunt for hydrocephalus and endocrine replacement was carried out in 2 elderly patients while it was carried out in 6 younger controls. Tumour biopsy and radical radiotherapy was carried in 2 younger controls while no such treatment was carried out in elderly patients. Endocrine replacement only was carried out in 06 elderly patients while no procedure was carried out in younger control. No specific treatment was carried out in four elderly patients. Chiasmal compression in elderly patients was successfully treated with trans-sphenoidal surgery, which was also well tolerated. The usual general anesthetic lasted around an hour, and patients were typically allowed to go home a week after surgery. The only long-term side effect of one older patient's surgery was a persistent impairment of vision, which may have been a symptom of pre-existing vascular illness. The majority of patients who had chiasmal compression surgery experienced useful improvement in vision.

A rare clinical syndrome known as pituitary tumour apoplexy (PA) is brought on by acute hemorrhage and/or infarction within a pituitary tumour that is often misdiagnosed [28]. Some authors examined in retrospect the occurrence, clinical manifestation, radiological and endo-crinological results, and surgical and medical treatment of pituitary apoplexy. They concluded that complete recovery is achievable even in the most severe cases provided appropriate management is started on time and a prompt diagnosis is made. In most cases, the surgical outcomes following the trans-sphenoidal approach are highly satisfactory [29-30]. It is reported that a patient who had a prior pituitary tumour resection but had a residual tumour underwent a successful caesarean section under anesthesia. During pregnancy, the pituitary gland experiences global hyperplasia. Enlargement may be a symptomatic feature of functional pituitary tumours during pregnancy. Acromegaly, which has anesthetic consequences such as challenging airway, systemic hypertension, diabetes, and electrolyte imbalance, is linked to growth hormone-secreting tumours. Lesions occupying intracranial space have the potential to cause herniation, lower cerebral perfusion, and raise intracranial pressure. A previous paper report this case's management [31].A previous case report represented successful management of pituitary enlargement in pregnant woman [32]. A case report presented diagnosis and management of pituitary tumour apoplexy following acute coronary syndrome management [33]. There was representation of successful operative management of recurrences of chromophobe pituitary tumour [34].

## Conclusion:

After the age of 70 years, 16% people having pituitary tumours were observed. The volume of fossa was statistically greater in elderly patients. The duration of follow up was statistically longer in younger controls. The visual defects observed in elderly group were greater than young patients. Pituitary adenomas in old patients can be treated with trans-sphenoidal-adenomectomy. However, the proportion is lower than younger controls. Post-operative radiotherapy was more commonly observed in old patients with pituitary adenoma than younger controls.

## Figures and Tables

**Table 1:** Details of patients and pituitary tumours

**Median(range)**	**Age at presentation (years)**	**Fossa volume (mm3)**	**Suprasellar extension (mm)**	**Follow-up (months)**
Elderly 20M, 16F	76 (71-83)	3291 (826-7841)	14 (0-28)	22 (2-44)
Younger 20M, 16F	52 (31-68)	2981 (1225-9051)	13 (0-26)	37 (7-68)
P value	0.001	0.001	0.07	0.001

**Table 2:** Presentation of pituitary tumors in elderly and young patients

	**Visual symptoms**	**Neurological symptoms**	**Endocrine symptoms**	**Incidental finding**	**Pituitary apoplexy**
Elderly (n= 36)	21	7	2	5	1
Young (n=36)	16	6	8	4	2
P value	0.001	0.67	0.001	0.89	0.9

**Table 3:** Treatment details for elderly and younger patients

	**Trans-sphenoidal-adenomectomy**	**Post-operative radiotherapy**	**Shunt for hydrocephalus and endocrine replacement**	**Tumour biopsy and radical radiotherapy**	**Endocrine replacement only**	**No specific treatment**
Elderly (n= 36)	24	2	2	0	6	4
Young (n=36)	30	0	6	2	0	0
P value	0.001	0.001	0.001	0.001	0.001	0.001
